# Fruit Development in Sweet Cherry

**DOI:** 10.3390/plants11121531

**Published:** 2022-06-07

**Authors:** Edoardo Vignati, Marzena Lipska, Jim M. Dunwell, Mario Caccamo, Andrew J. Simkin

**Affiliations:** 1 NIAB, New Road, East Malling ME19 6BJ, UK; edoardo.vignati@niab.com (E.V.); marzena.lipska@niab.com (M.L.); 2 School of Agriculture, Policy and Development, University of Reading, Whiteknights, Reading RG6 6EU, UK; j.m.dunwell@reading.ac.uk; 3 NIAB, Cambridge Crop Research, Lawrence Weaver Road, Cambridge CB3 0LE, UK; mario.caccamo@niab.com; 4 School of Biosciences, University of Kent, Canterbury CT2 7NJ, UK

**Keywords:** tree fruit, fruit ripening, rootstock, *Prunus avium*

## Abstract

Fruits are an important source of vitamins, minerals and nutrients in the human diet. They also contain several compounds of nutraceutical importance that have significant antioxidant and anti-inflammatory roles, which can protect the consumer from diseases, such as cancer, and cardiovascular disease as well as having roles in reducing the build-up of LDL-cholesterol in blood plasma and generally reduce the risks of disease and age-related decline in health. Cherries contain high concentrations of bioactive compounds and minerals, including calcium, phosphorous, potassium and magnesium, and it is, therefore, unsurprising that cherry consumption has a positive impact on health. This review highlights the development of sweet cherry fruit, the health benefits of cherry consumption, and the options for increasing consumer acceptance and consumption.

## 1. Introduction

Cherry (*Prunus avium*) is believed to be native to Europe and southern Asia [1] and to a small isolated region in the western Himalayas. Commonly called sweet cherry, it is a deciduous tree, 15–32 m in height and with a trunk up to 1.5 m in circumference. It belongs to the Rosaceae family, which includes many plants, such as rose, apple (*Malus x domestica*), peach (*Prunus persica*) and strawberry (*Fragaria vesca* and *F. ananassa)* that are very important for the human economy. The sweet cherry genome is diploid (2n = 16), simple, compact and about 350 Mb in size. The genome structure is predicted to be similar to that of the peach, although the sequences have diverged [2,3,4] See (http://www.rosaceae.org accessed on 1 June 2022) [5].

Young cherry trees show strong apical dominance with a straight trunk and symmetrical conical crown that becomes rounded to irregular for old trees. All parts of the plant, except for the ripe fruit, are slightly toxic, due to the presence of cyanogenic glycosides. The species has also become naturalized in North America and Australia since it is largely cultivated in these regions.

**Figure 1:**
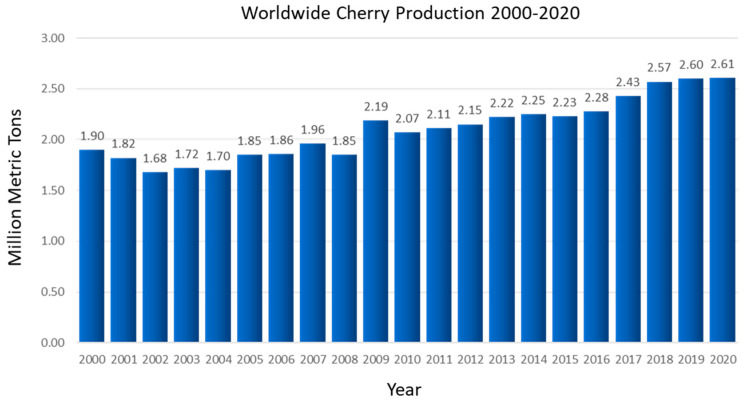
Graphical representation of worldwide cherry production between 2000 and 2020 (Statista.org).

Cherry production has seen remarkable growth in the last few years. Worldwide, cherry production has increased by 37% since 2000 (Figure 1). This fact shows that growers are committing more resources to cherry production, and growing consumer interest is one of the drivers behind these increases.

## 2. Cherry from Flower to Fruit

### 2.1. Cherry Flower

#### 2.1.1. Cherry Flower Pollination

The sweet cherry flower is hermaphroditic; possessing both female and male reproductive organs. Each flower is approximately 2.5 cm in diameter, with five petals surrounded by five green sepals, a single upright pistil with an ovary, two ovules, and 30 stamens. The stamens are the male reproductive organs consisting of anthers, where the pollen develops; it sits on top of the stalk-like filaments, which allows water and nutrients to reach the anthers from the mother plant and facilitate pollen dispersal [6,7]. The pistil or gynoecium is the female reproductive organ, and it occupies the centre of the flower.

The flowers open for between three and five days and the stigma is receptive to pollination at this time. The anthers begin to open shortly after the flower and continue into the second day [8]. In sweet cherry, it takes two to three days for the pollen tube to grow from the stigma to the base of the style, whilst fertilization occurs six to eight days after pollination [9,10]. Stösser and Anvari [11] determined that effective pollination takes between 4 and 5 days; however, under some conditions, effective pollination can last up to 13 days [12]. There are a number of varieties of sweet cherry that exhibit self-fertility, the first and most notable being ‘Stella’, which was first identified in Canada [13], and is highly sought-after and considered a cultivar of great importance [14,15,16]. However, most sweet cherry cultivars exhibit self-incompatibility (SI), a characteristic that involves inhibition of pollen tube growth, and is genetically controlled by multiple allelic *S*-loci [17,18,19,20]. SI sweet cherry varieties still produce a small number of fruit through self-pollination; however, different varieties are considered to have different levels of self-sterility, with important commercial varieties, such as ‘Kordia’ and ‘Regina’ being highly incompatible and setting virtually no fruit in the absence of cross-pollination [21].

The nectar of sweet cherry is rich in sugars (from 28% to 55% sugar), the most abundant of which are fructose, glucose and sucrose, which are highly attractive to pollinating insects, including bees [22]. It is well known that insect-mediated pollination in sweet cherry is important for the production of a viable crop and besides the environmental conditions, pollinating insects are the most important factor governing yield [23]. Work by Holzschuch et al. [24] showed that bagged flowers produced only 3% of the number of fruits produced by unbagged flowers and that the rates of pollination and fruit set were related to wild bee visitation. Wild pollinators, including solitary bees, have been described as being instrumental in achieving adequate sweet cherry yields [25]. These authors also showed that pollination by wild bees surpassed pollination by honeybees. However, even with this knowledge, less than 17% of growers provide trap nests for solitary bees in their orchards and rely on commercial domesticated honeybees (*Apis mellifera*) to carry out this function at a cost up to 1000 Euro per hectare—a considerable investment for commercial cherry producers. A recent review has outlined grower knowledge of the role of insects on sweet cherry crops [23].

Air temperature is also known to influence flowering and fruit sets and has no influence on the level of SI; however, the air temperature may influence pollen tube growth [26]. The optimal temperature for flowering has been reported to be around 20 °C, with temperatures as low as 15 °C slowing the disappearance of the embryo sac, as compared to 25 °C [27]. Temperatures exceeding 30 °C are considered too high for successful flowering [26,28,29]. Furthermore, it has been reported that a sudden fall in temperature at the end of the flowering period can result in a total loss of the crop [21].

#### 2.1.2. The ABCDE and the Floral Quartet Models

**Figure 2:**
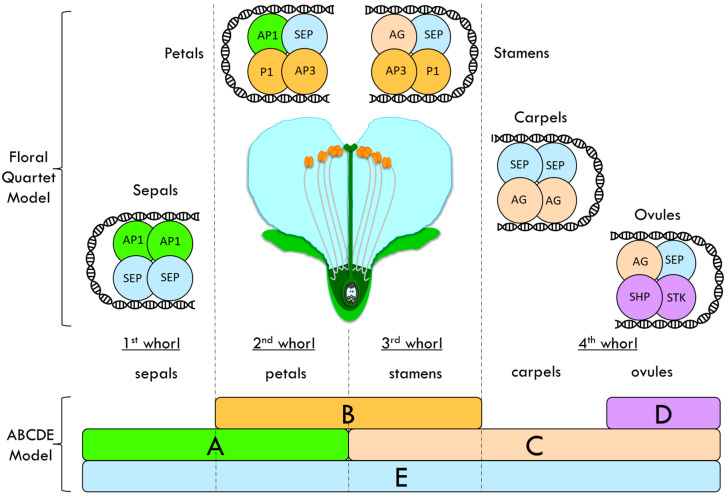
Floral quartet model (FQM) integrated into the ABCDE model proposed to explain the flower whorls’ identity determination in *Arabidopsis thaliana*. This enhanced ABCDE model postulates that sepals are specified by A + E, petals by A + B + E, stamens by B + C + E, carpels by C + E and ovules by C + D + E. Class-A protein: APETALA1 (AP1); Class-B proteins: PISTILLATA (PI) and APETALA3 (AP3); Class-C protein: AGAMOUS (AG); Class-D proteins: SEEDSTICK (STK) and SHATTERPROOF (SHP); Class-E protein: SEPALLATA (SEP).

Flower organs are arranged in whorls, with sepals that are located in the most external whorl and carpels in the inner whorl, in the form of petals and stamens. Floral homeotic mutants, i.e., mutants with an altered floral organ identity, such as in *Arabidopsis thaliana* and *Antirrhinum majus* (snapdragon), have been studied and used to propose the ABC model of flower formation [30,31]. ABC genes encode three different classes of MADS-domain transcription factors involved in the flower organ identity determination. These factors interact to give the organ identity to sepal, petals, stamens and carpel. Further studies showed the existence of two other classes: the D class, which is responsible for the ovule identity, and the E-class, which has been proposed as another class of redundant floral organ identity genes [32]. This enhanced ABCDE model postulates that sepals are specified by A + E, petals by A + B + E, stamens by B + C + E, carpels by C + E and ovules by C + D + E (Figure 2) [32,33,34,35].

In addition, the floral quartet model (FQM, Figure 2) has been suggested to integrate the ABCDE model [32]. According to the FQM, the factors form tetrameric complexes to carry out their functions, binding the DNA to activate or repress the expression of their target genes [32]. In particular, two MIKC-type MADS-box transcription factors belonging to the same class form a dimer, which can interact with another dimer of the same class or of a different class, to promote the development of a specific floral organ [35]. The MIKC-type MADS-box transcription factors are composed of specific domains:

1. MADS (M) domain: a DNA-binding domain, but it is also involved in dimerization and in nuclear localization. It is the most conserved domain among the MADS-box transcription factors [36].
2. Intervening (I) domain: takes part in the selective formation of DNA-binding dimers. It shows only limited conservation [37].
3. Keratin-like (K) domain: an essential element for dimerization and multimeric complex formation. This domain has a particular structural organization (amphipathic helices) in which the hydrophobic and charged residues are conserved and regularly spaced [38,39].
4. C-terminal (C) domain: is somewhat variable. In some cases, it takes part in the transcriptional activation of the target genes or in the formation of multimeric complexes [37].

According to the FQM, MADS-box transcription factors form dimers, which bind the DNA where the CArG-boxes are present. The two dimers that are present in the tetramer recognize and bind the two different CArG-boxes—which can be far away in the DNA sequence—by looping the DNA and making them spatially close [32,35,40].

## 3. Cherry Fruit Development

### 3.1. Cherry Fruit

The sweet cherry fruit is a drupe—an indehiscent fruit of 1–2 cm in diameter (in some cultivars the diameter can be larger) that has an attractive appearance due to its color (bright red to dark purple depending on the cultivar) and desirable, intense flavor. Three parts can be identified in a drupe: the outer exocarp or skin; the mesocarp, which is the fleshy part of the fruit; and a single central stone, which is the lignified endocarp that surrounds the seed. Drupe development in cherry fruit is consistent with the reported stages of growth in other drupes, as described below.

Cherry varieties can be divided into early, mid and late ripening types (Table 1) based on the ripening of the reference variety at the European level, Burlat, a widespread cultivated cherry across Europe.

During early fruit development, there is constant communication between the developing fruit and the developing seed. These signals can be hormones, such as auxin and gibberellins, produced in the endosperm or in the seed coat and are involved in the developmental synchronization of all these different structures [41,42,43,44]. The disruption of this communication is one of the triggers of fruit drop—the loss of a significant proportion of their fruit before ripening. This loss, which is often referred to as ‘June Drop’ or ‘Cherry Run Off’, varies from year to year and, in some seasons, can result in a total loss of the crop.

### 3.2. Physiological Changes during Cherry Fruit Development

**Figure 3:**
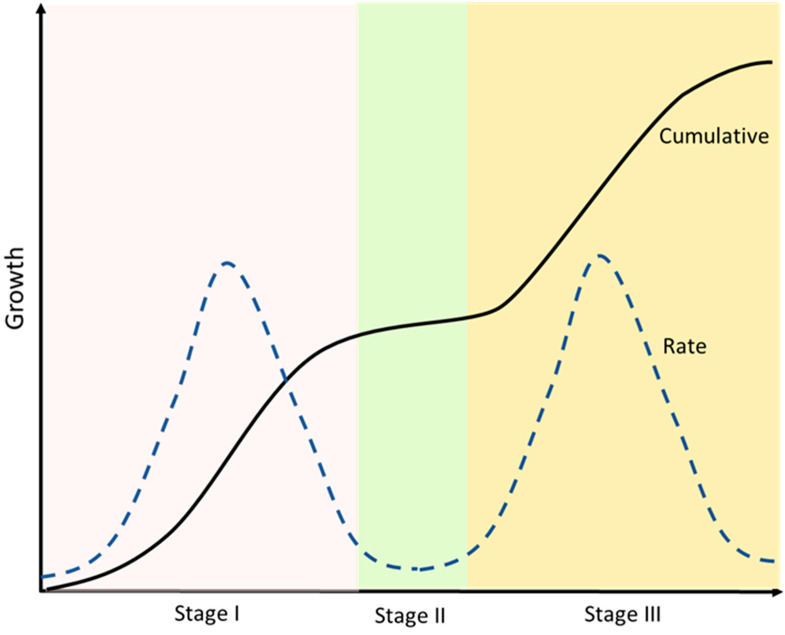
A double sigmoid growth curve representation. Cumulative and rate of growth are defined. After an initial exponential phase (Stage I) a second plateau phase occurs (Stage II), which is followed then by a second exponential phase (Stage III) until the complete ripening of the fruit. Developmental phases are allocated with respect to peaks and troughs in growth rate.

The developmental growth rate of the drupe can be described by a double sigmoid curve, where three well-marked stages can be identified (Figure 3). In the first phase (stage I), the ovary either aborts or starts all the subsequent morphological changes that will lead to the formation of the fruit [45,46,47,48,49,50,51]. The traditional definition of phase I, beginning at anthesis, also includes the acceleration of the growth of the ovary approximately two weeks before anthesis [52].

**Figure 4:**
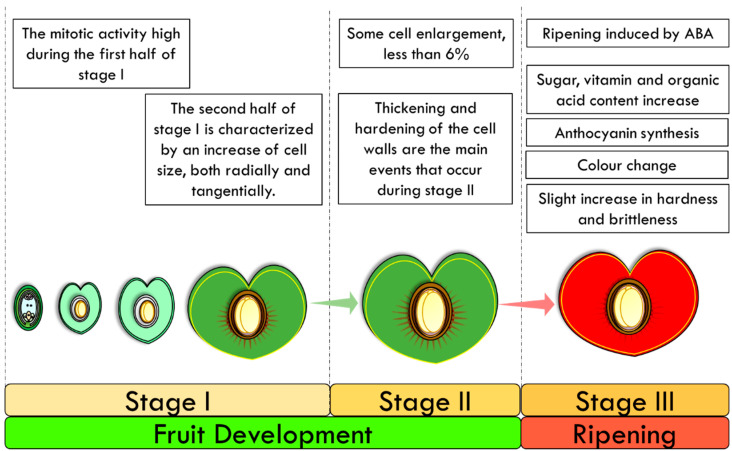
Fruit Ripening in the cultivated cherry. After an initial exponential phase (Stage I) a plateau phase occurs (Stage II), which is followed then by a second exponential phase (Stage III) until the complete ripening of the fruit.

The outer epidermis of the developing cherry fruit consists of a single row of cells that are covered externally by a cuticle, which is interrupted only where the stomata are present (see Section 4). During the pre-flowering period, the cells of the epidermis are already well-differentiated, with an elongated shape and no hairs, and increase their size slowly, mainly in the radial direction. The mitotic activity is low since the increase in the number of cells is very limited, while it is high during the first half of stage I (Figure 4). The second half of stage I is characterized by an increase in cell size, both radially and tangentially. Moreover, during the second half of stage I, the cuticle reaches its full thickness. No cell division is observed during stage II, which is characterized by slight tangential elongation of cells and additional cell wall thickening. Lastly, during the final phase (stage III), cells start to enlarge by as much as 100-fold, contributing to the final fruit size. After these three phases, a green immature fruit is formed, which has the dimensions of the mature fruit and starts the maturation process (see Section 3.3) [45,46,47,48,49,50,51]. A more than two-fold tangential enlargement of the cells occurs, with a decrease in the radial diameter and cuticle thickness, in order to generate a large increase in the fruit surface area [53].

During the pre-bloom period, the fleshy mesocarp is composed of isodiametric parenchymatous cells, which elongate according to a gradient tangentially to slightly radial from the epidermis to the stony endocarp, respectively. The increase in tissue thickness, more than double, is due mostly to cell division. The first half of stage I is characterized by cell mitotic divisions, accomplishing an increase of about 20–30% in the number of cells from the epidermis to the stone, and no further divisions are observed after completion of this stage [53]. The cells increase in diameter more than four-fold, mostly during the second half of stage I. Moreover, differentiation of a hypodermal layer, five or six cells thick, occurs just under the epidermis, by considerable thickening of the cells. These cells become slightly greater in size than those of the epidermis and become tangentially elongated. On the other side, adjacent to the stone, a layer of small isodiametric cells, three or four cells wide, differentiates, during the second half of stage I. During stage II, some cell enlargement, less than 6%, occurs and intercellular spaces remain prominent. The cells just under the epidermis continue slowly to elongate tangentially, while limited cell division can be found in the thin layer of small cells adjacent to the stone, otherwise, there is none. Stage III is also called “the final swell” and during this period, four parts of the fleshy mesocarp can be recognized (from the epidermis to the stony endocarp):

1. The hypodermal layer of collenchyma;
2. The peripheral layer of thin-walled parenchyma, extending to a line just inside the ring of vascular bundles;
3. The layer of radially elongated cells, extending from this line nearly to the stone;
4. The thin layer of small cells adjacent to the stone.

The transition from one layer to the other is not precise, since there is a gradual transition from a tissue in which one cell type predominates to one in which another one is dominant. A gradient of cell elongation can be identified, from tangential in the hypodermal cells to radial in the ones close to the stone. The rapid cell enlargement brings a decrease in the thickness of the cell walls and the disappearance of the intercellular spaces that are now difficult to identify [53].

The ventral and the dorsal capillary bundles do not enter the stony endocarp, while the two bundles that supply the two ovules (in peach called “funicular bundles”) are at either side of the ventral suture and adjacent to the ovarian cavity. Pit bundles traversing the stony endocarp, of the type found in peach, are not present in cherry. During the pre-bloom period, the differentiation of a ring of vascular bundles, which extends through the fleshy mesocarp parallel to the central axis, occurs. However, the walls of xylem elements do not become sufficiently thickened to become conspicuous until about the full-bloom period. The number of bundles is fixed, so that as the fruit develops, the vasculature system extends progressively throughout the flesh, mostly by a transverse divergence of small bundles from the main ones. At maturity, a skeleton network of conductive tissue is present [53].

The innermost ovary wall comprises a single layer of cells, called the inner epidermis, and three or four layers of pericarp cells. These cells, which are going to become part of the stony endocarp, are smaller than the cells that are going to become the fleshy mesocarp, and this size difference persists to maturity. During the pre-bloom period, the inner epidermal cells elongate tangentially and divide transversally just before full bloom, to form with adjacent pericarp cells with a similar orientation—a “hoop” shape that surrounds the ovary cavity. The presence of this hoop is also reported in other species [54,55]. The group of cells that differentiate into the stony endocarp do not give rise to the fleshy mesocarp and vice versa. Stage I is characterized by a large increase in both the number and size of the inner layer cells (tangential elongation) and the outer layer cells (central radial elongation). No cell divisions are observed in the inner layer during the second half of stage I, while there is a greater increase in the mitotic divisions and cell size in the outer layer. Cell walls become progressively thicker but do not harden significantly until the end of this stage, which is when the pit cannot be sectioned without special treatment [53]. The thickening and hardening of the cell walls are the main events that occur during stage II, and no cell divisions are observed after the mid-point of this stage. Hardening does not occur uniformly in the stony endocarp, but it begins near the apical end at the hilum, and continues downwards toward the chalaza before finally including the whole endocarp. Although lignification occurs, cells are living and nuclei are abundant, even though the cellular lumen is frequently scarcely larger than the nucellus. At the end of stage II, the stony endocarp is made of 26–27 layers of very thick-walled lignified cells. This number of layers is the same as that present in the fleshy mesocarp, although the thickness is much less due to the very different destinies of the two tissues. During stage III, there is a slight increase in hardness and brittleness that is associated with a loss of moisture. It is possible to find occasional living cells with active nuclei [53].

### 3.3. Final Ripening Stages of Mature Cherry

**Figure 5:**
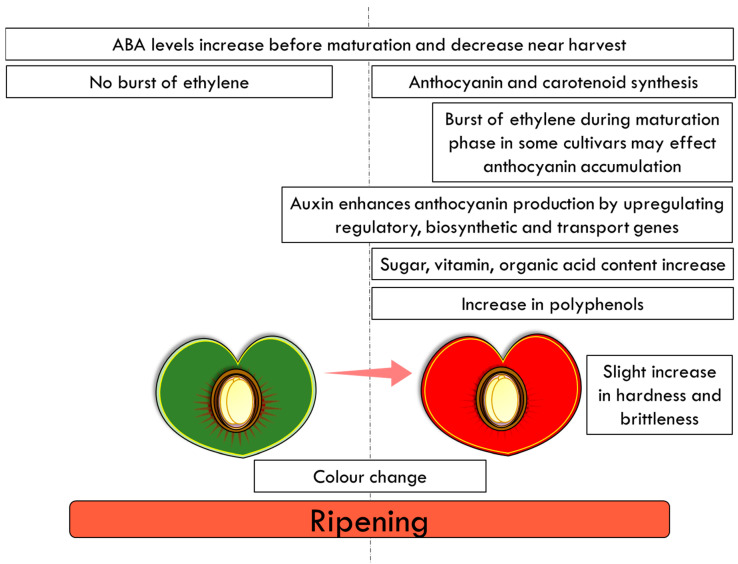
Final stages of fruit ripening in the cultivated cherry.

Fruit ripening is a highly variable process with numerous differences when different plant species are compared; however ripening is often characterized by the accumulation of pigments, such as carotenoid sand anthocyanins (Figure 5) [56]. Despite these differences, fruit ripening has been classified into two distinct groups, climacteric and non-climacteric, which differ in their patterns of ethylene production and respiration at the onset of the ripening process [57,58]. Climacteric fruit, such as tomato, display a rise in respiration and a burst of ethylene modulating the differentiation of chloroplast to chromoplast and the accumulation of carotenoids [59,60]. In contrast, cherry fruit are non-climacteric and ripening is promoted by abscisic acid (ABA) [61]. No burst of ethylene and respiration has been observed in sweet cherry during fruit development and ripening. Moreover, the exogenous ethylene treatment has no significant effects on the fruit’s respiration rate or on its softening [62,63]. Ethylene concentrations remain low [64,65], although ethylene may affect the accumulation of anthocyanins [66]. However, it should be noted that the ethylene concentration in some sweet cherry cultivars has been shown to increase sharply before harvest [67], suggesting that it may play some role in fruit ripening during the maturation phase of development [68]. In sweet cherry, ABA levels increase before maturation and decrease near harvest [64,69]. Furthermore, in grapes, another non-climacteric fruit, the onset of ripening is ABA related; ABA modulates the changes in color and the accumulation of sugars and has a role in fruit softening in the later stages of ripening [70,71,72].

Cherry ripening is associated with changes in color, sugar, vitamin and organic acid content (Figure 5). In sweet cherries, ABA has been associated with the regulation of anthocyanin synthesis and the organoleptic properties (ratio of total soluble sugars to total acidity) [64,66,73]. The color of the fruit is attributed to the accumulation of these water-soluble health-promoting anthocyanins (a class of flavonoids), which are responsible for the blue, purple and red colors [74,75]. In addition to ABA, cytokinins, jasmonic acid (JA), gibberellins (GAs), and auxins, all play roles in the ripening of non-climacteric fruit. Auxin (1-naphthaleneacetic acid (NAA)) treatment has been shown to enhance anthocyanin production in sweet cherry by upregulating key anthocyanin regulatory, biosynthetic and transport genes [76]. This study demonstrated that treatment with NAA alters ethylene production, induces ripening, and enhances anthocyanin production, probably through ABA metabolism [76].

Size, color, firmness, sweetness and flavor intensity are considered the most critical attributes that drive consumer acceptance and, ultimately, the value of the crop to the producers [77,78,79,80]. However, consumers from different countries and regions place different values on their requirements for a good cherry. For example, consumers in Norway prefer dark, large cherries [81,82]. Similar results were found in the UK [83] and in American markets [84]. These consumer requirements may differ from those of the growers and supermarkets. Growers want cherries that are resistant to cracking and can be left on the trees longer to ripen, are easy to harvest (long peduncles) and firm enough to resist damage during picking, processing, and transporting. Supermarkets want cherries with long shelf lives, even weeks once in store.

## 4. The Physiological Role of Cherry Fruit Stomata

**Figure 6:**
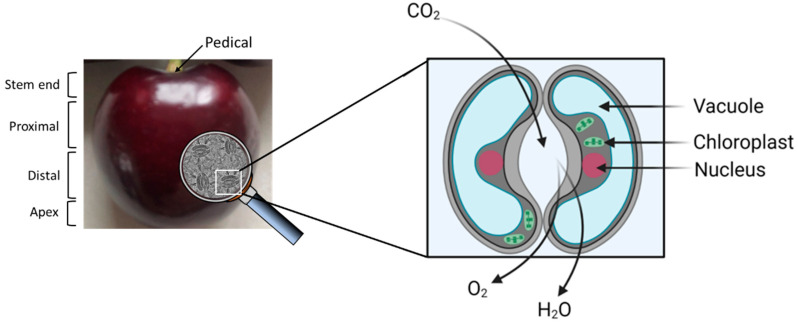
Schematic representation of stomatal pores on the surface of cherry fruit. They are found in high numbers near the apex and few near the stem end. When the stomata open, Ribulose–1,5-bisphosphate carboxylase (Rubisco) assimilates atmospheric CO_2_ that diffuses into the cells through the stomatal pores. Simultaneously, water and oxygen are lost (Figure created using Biorender.com 1 June 2022).

Although stomata are routinely found on the surface of cherry fruit [85], they are not uniformly distributed over the surface of the epicarp (Figure 6). They are found in high numbers near the apex and few are near the stem end.

The full differentiation of stomata can already be found during the pre-bloom period and the guard cells increase in size as the fruit develops, but at a lower rate than the epidermal cells. In the ripe fruit, the cells of the epidermis have a bright red color due to the presence of the chromoplasts, which are absent in the guard cells and in the cells that are immediately adjacent, resulting in a yellowish, straw-color [53]. Studies that have evaluated photosynthesis in non-foliar tissue, including fruit, have assumed that the photosynthetic pathway is similar to that observed in the mesophyll. However, non-foliar tissue photosynthesis has two potential major sources of CO_2_. Firstly, Ribulose–1,5-bisphosphate carboxylase (Rubisco) assimilates atmospheric CO_2_ that diffuses into the cells through the stomatal pores and, secondly, CO_2_, released by mitochondrial respiration, is re-fixed (recycling photosynthesis) [86,87].

The fruit of cucumber (Cucumis sativus) was shown to contribute 9.4% of its own carbon requirements, and as much as 88% of the respiratory CO_2_ in fruit was re-fixed [88].

Tomato fruits also have no stomata on their surface [89,90]; however, work carried out by Tanaka et al. [91], Hetheringon et al. [92] and Obiadalla-Ali et al. [93] in green developing tomato fruit showed that fruit contributed between 10% and 15% of the total fixed carbon of the fruit. Similar to the tomato, cherry fruit are green at the early stages of development and contain the necessary pigments to carry out photosynthesis (for a review of photosynthetic pigments and fruit photosynthesis, see [86,89,94,95]. 

These data suggest that the stomata on the surface of cherry fruit carry out a real physiological role, contributing carbon either directly by assimilating carbon from the atmosphere or through recycling photosynthesis, recapturing the CO_2_ liberated by mitochondrial respiration. A better understanding of this process could lead to better quality and a more nutritious product.

## 5. Genetic Control of Fruit Development

Fruit development is tightly controlled and regulated in order to produce, in the end, the complex structure that bares the developing seed. There are several models used to explain this complicated process, however, the most commonly used is the silique of *A. thaliana*, which is a dry fruit made from several distinct tissues, including the valves (seedpod walls), replum (middle ridge), septum, and valve margins [96], and tomato (*Solanum lycopersicum*), which has become the primary model to study fleshy fruit development and ripening because of the availability of advanced molecular and genetic toolkits, mutant collections and ease of handling [59,97].

The features of an organ are determined at a very early stage of development. During the very first phases of development, many aspects of floral organs, such as ovary identity and size, and at least partially final fruit size and ripening, are determined. Therefore, it is not surprising that some transcription factors involved in flower organ determination are also involved in fruit development.

The C-clade MADS-box transcription factor iAGAMOUS (AG), is involved in stamen, carpel and ovule identity determination [98]. AG is also expressed both in the stamens and in the carpels of peach. The PpeAG sequence present in GenBank (accession AAU29513) was used for the sequence alignment. PpeAG is also expressed at high levels in the mesocarp and the endocarp during fruit development [99]. In tomato, the downregulation of TOMATO AGAMOUS 1 (TAG1) by RNAi not only causes flower defects, such as stamen with petaloid tissue, but also leads to a loss of flower and fruit determinacy, producing flower-in-flower and fruit-in-fruit phenotypes [100,101]. The important role of AG in both flower and fruit development is reported in other species, such as strawberry (Fragaria × ananassa), cocoa (*Theobroma cacao* L.) and grape (*Vitis vinifera* L.), which produce different types of fruit [102,103,104].

Another gene involved in the post-fertilization differentiation of fruit tissues is FRUITFULL (FUL). This gene encodes a MADS-box transcription factor that negatively regulates the valve-margin identity genes in *A. thaliana* [105], and its expression is first observed in the carpel primordia during early flower development and later only in the valves’ regions. Targets of AtFUL are the SHATTERPROOF1 (SHP1) and SHATTERPROOF2 (SHP2) genes. The AtSHP genes also encode the MADS-box transcription factors, which are necessary for the differentiation of the dehiscence zone, at the valve margin close to the replum, and for the lignification of the neighboring cells. AtSHP1 and AtSHP2 are involved at the beginning of the genetic pathway that regulates the formation of the dehiscence zone [106]. These genes are also important in tomato fruit development. Indeed, a reduction in SlFUL1 and SlFUL2, which are the homologs of AtFUL, greatly affects late fruit development, impacting the ethylene-independent ripening process [107,108,109]. Moreover, knock-down of the tomato SHP orthologue, TOMATO AGAMOUS-LIKE1 (TAGL1), affects both the flower and fruit development, causing a loss of the style trichomes and a thinner fruit pericarp, respectively [110], while its ectopic expression leads to the development of lycopene-rich fleshy sepals, supporting the typical function of the C-type genes [100,111]. Finally, the combination of these mutant phenotypes and the gene expression patterns during fruit development seems to support the model of a negative regulation of TAGL1 by SlFUL1 and SlFUL2, also in tomato, thus, confirming the conservation of their roles and their regulatory interactions. This model is further supported by the results found in peach (*Prunus persica*). Whilst very little is known about the genetic regulation that occurs during the differentiation of the cherry fruit in all its components, many studies have been carried out in peach, which is emerging as a plant model for tree species, thanks to its small genome size of 300 Mb, which is slightly smaller than that of cherry (350 Mb), and about twice that of *A. thaliana* (135 Mb), and with a moderately short juvenile phase of 2–3 years.

Peach, like cherry, belongs to the same clade of Rosids as Arabidopsis and they share anatomical and physiological similarities regarding their flowers and fruits. In the peach genome, one putative PpeFUL ortholog gene and two copies of the PpeSHP genes have been identified. Once aligned to the closest matching homologous protein sequences, both PpeFUL and PpeSHP showed the MADS domain are conserved, along with the conserved regions in the I and K domains, while the C-terminal domain was observed to be more divergent in both cases [112]. In addition, PpeSHP has been shown to maintain its typical function as a C-type MADS-box transcription, since once overexpressed in tomato, it promotes the formation of carpel-like fleshy and reddish sepals [113]. PpeFUL is expressed mostly in the mesocarp and in the exocarp, while its expression is very low in the endocarp. In contrast, PpeSHP gene expression is high in the endocarp but almost zero in the mesocarp and in the exocarp [114]. Furthermore, peach cultivars with the split-pit phenotype, which is associated with a higher degree of lignification, show higher expression of PpSHP. Interestingly, the split-pit is a structure similar to the dehiscence zone of the dry fruits, where the important role of FUL and SHP in regulating lignin deposition, cell expansion and cell separation processes have been demonstrated [106,112,115,116]. Thus, all these results suggest that this regulatory pathway, where FUL is responsible for the inhibition of SHP, which in turn, is upstream in the lignification pathway and seems to be conserved in dry and fleshy fruits, at least in the core eudicots.

Lastly, SHP1 and SHP2 are found to be associated with a D-class transcription factor, SEEDSTICK (STK). In Arabidopsis, STK is not only involved in the differentiation within the endocarp of the layer that will lignify upon maturation but is also a master regulatory gene of ovule development, since ectopic expression of STK is sufficient to induce the formation of ectopic ovules [117]. On the other hand, one of the most evident phenotypes observed in the stk single mutant is the failure of the seed release from the mature fruit, due to a lack of lignification [118]. In peach, one putative copy of the STK gene was found and comparative analysis showed that it belongs to the D lineage along with the other D-type MADS-box transcription factors genes and that the typical D-lineage sequences at the C-terminus and the extension between the stop codon and the AG motif II are conserved [99]. PpeSTK expression is very high in the endocarp at the beginning of the fruit development, and it decreases progressively during the following weeks, while it remains low both in the exocarp and in the mesocarp [114]. Therefore, it seems that there is a spatial regulation of gene expression during peach fruit development, and it could resemble the expression pattern observed in Arabidopsis. In conclusion, STK seems to be involved in the specification of the endocarp, since another example is reported in the fruit of oil palm where the natural stk mutant is almost stoneless [119].

## 6. The Accumulation of Bioactive Compounds during Ripening

Sweet cherry fruits are nutrient-dense, high in fibre and accumulate a number of bioactive compounds, including, carotenoids, polyphenols, anthocyanins, vitamin C and potassium, during ripening [120,121]. (For a review, see Ansari and Emami, [122]; Aloum et al. [123]; Moshiri et al. [124], Milani et al. [125], Faienza et al. [126], Chockchaisawasdee et al. [127] and Pashirzad et al. [128]). In cherry fruit, polyphenols are concentrated in the skin and anthocyanins, cyanidin 3-glucoside, cyanidin 3-rutinoside, cyanidin 3-sophoroside, pelargonidin 3-glucoside, pelargonidin 3-rutinoside, 3-glucoside, and peonidin 3-rutinoside, have also been identified [129,130,131]. Cyanidin 3-O-glucoside has been shown to possess strong antioxidant activity [132] and to have a positive effect on liver ischemia-reperfusion injury (preventing cell death following the restoration of blood flow) [133]. Human trials have suggested that anthocyanins slow glucose production from more complex carbohydrates, promoting healthy glucose regulation and decreasing the likelihood of type-2 diabetes [134].

Given the high concentrations of bioactive compounds (carotenoids, polyphenols anthocyanins, etc.) in cherries, it is not surprising that their consumption has a positive impact on health. These data indicate that the regular consumption of fruits promotes health and reduces the risks of disease and age-related decline in health [135,136,137,138,139,140].

Published trials on animal and human subjects have suggested that cherry fruit consumption may reduce the risk of several chronic inflammatory diseases (i.e., arthritis, diabetes), and improve sleep and cognitive function. In the last few years, cherries have also generated great interest because of their health benefits, the subject of several reviews [120,121,141,142,143,144]. These data suggest that increased cherry consumption may improve the health and quality of life of consumers. Identifying a means of generating a seedless, stoneless cherry has the potential to make the fruit even more attractive to the consumer. Previous data has shown that seedless cultivars of fruit, such as sweet orange (*C. sinensis*), grape (*V. vinifera*), and watermelon (*Citrullus lanatus*) have significantly increased consumer consumption [145].

## 7. Cherry Tree Cultivation, Environment and Tree Management 

### 7.1. Climate Impacts on Cherry Tree Cultivation and Yield

It is important to note that climate plays an important part in cherry tree cultivation and final crop yield. With global increases in temperature of 2 °C, an increase in atmospheric [CO_2_] from 420 ppm to 550 ppm by 2050 (the former already elevated compared to pre-industrial values (278 ppm) and 700 ppm by 2100 [146,147,148,149,150], and an increase in rainfall in some areas and a reduction in rainfall in others, it is important to understand how these environmental conditions will affect cherry tree cultivation. To determine the role of atmospheric changes on cherry tree development, sweet clonal cherry (*Prunus avium* L.) plants were propagated for 19 months under glass at ambient (1994–358 ppm; 1995–360 ppm) or elevated [CO_2_] (*e*[CO_2_) (700 ppm—expected by 2100). Initially, in (*e*[CO_2_), photosynthesis increased and a concomitant increase in dry matter (leaf—55% and stem −61%) was observed after two months of growth, suggesting that increasing [CO_2_] could be beneficial for cherry tree cultivation. However, after 10 months, photosynthetic rates decreased, and only small increases in dry mass were still evident, which suggests sweet cherry acclimates to *e*[CO_2_] due to long-term exposure [151]. Due to the young nature of the plants studied, no information is available to determine the impacts of *e*[CO_2_] on fruit yield or quality, however, these results suggest that cherry trees may not benefit significantly from increased atmospheric [CO_2_].

In addition, future climates may also encounter increased rainfall, which can result in rain-induced cracking, a major cause of crop loss in sweet cherry production in many regions of the world [152]. Cracking is thought to be induced by water uptake on the surface of the fruit, and via the root system (heavy rain during the harvest period), resulting in swelling of the epidermis, localised bursting of the cells releasing malic acid, and a weakening of the epidermal and hypodermal cells until finally, cracking occurs (for a review, see Carreia et al. [152]). This may become more of a problem in regions where increased rainfall is observed due to climate change. Currently, pre-harvest foliar treatment with calcium, in combination with gibberellic acid, is used to mitigate cracking due to an increased water uptake [153,154].

In other regions, a reduction in rainfall is expected. Water availability influences growth [155,156], yield and fruit quality characteristics of sweet cherry [157]. Water is an important requirement in cherry fruit production and irrigation is essential in areas where rainfall is limited (areas likely to increase in future years). A reduction in rainfall in some areas will increase the reliance on irrigation and the use of groundwater for crop production, which may also be limiting. Cherry production may not be seen as a priority in areas of low rainfall, where water is required to irrigate crops of greater importance as the population increases, and the need for higher calorific crops, such as wheat and maize, are prioritized. Changes in rainfall may also change soil moisture and soil temperature. Under these circumstances, a change in rootstock might be required to successfully propagate cherries (see Section 7.4).

Recent changes in climate have also resulted in a requirement to protect crops against extreme environments, heavy rain and hail, and have resulted in the introduction of new protected growing environments, such as polytunnels (see Section 7.5) [158].

### 7.2. The Use of Hormone and Biostimulant in Cherry Cultivation

Many temperate woody plants, including cherry, possess a dormancy cycle to prevent premature bud bursts during short periods of warmer weather, which are then subsequently followed by colder weather, thus causing shoot damage (see below) [159]. Following a period of exposure to low temperatures, dormancy is broken, and bud growth is initiated, following a return to favorable growing conditions [160,161,162,163]. Some varieties, such as Bing (Table 1), require up to 700 h at temperatures below 7 °C to break dormancy. In the absence of sufficient chill hours, seen more often due to rising global temperatures, with an expected mean global temperature increase of 2 °C by 2050 [164], cherry yields can be considerably reduced [163]. There are currently several agents on the market that synchronize fruit ripening following application in the absence of required chill hours. One of these reagents, hydrogen cyanamide, has been associated with an increase in flower numbers per shoot [165,166,167,168]. ERGER^®^ is a biostimulant used to promote uniform bud break and has been shown to break dormancy in axillary and terminal buds in a number of woody species, including apples [169].

At a later stage of development, cherry trees are often treated with plant hormones such as gibberellic acid to stimulate fruit growth and increase fruit firmness [170,171]. A recent publication by Basile et al. [172] evaluated the use of biostimulants (tropical-plant extract) on two sweet cherry varieties, Kordia and Regina (Table 1). In both cultivars, foliar application of this extract induced significant increases in fruit yield. In Kordia, treatment was also shown to improve fruit quality (enhanced fruit calcium concentration by 26.2%, soluble solids content by 11.8%, increased flesh firmness, and skin color by 6.7%, and 12.0%, respectively) [172]. These authors have suggested that biostimulants could be a sustainable and effective alternative to the exogenous application of synthetic hormones and nutrients currently used in cherry cultivation.

### 7.3. Thinning and the Environment

Many trees produce a large quantity of flowers, and as observed in apples and pears, the trees thin themselves, so as to not overburden the plant and reduce the chance that branches will break under the weight. This process, referred to as ‘June Drop’, begins in late June until mid-July, with the expectation that just one in twenty flowers produce fruit. However, cherry trees often do not drop flowers, and thus require additional resources and mechanical and hormonal thinning to protect the yield and quality. In the past, the requirement for thinning during cherry production was rare; however, due to the introduction of dwarfing rootstock (see Section 7.4), thinning has become necessary to avoid excessive flowering [173]. Thinning can be carried out via large-scale pruning, mechanical hand removal of flowers or chemical thinning. These different methods have recently been reviewed by Rutkowski and Lysiak [173]. Thinning, however, comes with inherent risks. Firstly, thinning increases the size of the remaining fruit, which also increases cracking susceptibility [174], and importantly, this can reduce the harvest if a late frost occurs after thinning. For example, the process, ‘June drop’ can be triggered by unfavorable environmental conditions. A late-spring frost may damage bud tissue, and lead to cherry trees losing a fraction of their fruit before ripening [175], which, if this occurs after thinning, can result in a significant loss of yield. This loss can vary from year to year. In 2020, in the UK, late frosts resulted in a 90% loss of fruit before harvest.

### 7.4. Cherry Rootstock

Traditional cherry trees can be challenging when grown on their own rootstock due to their large size. Cherry trees also take up a large amount of space and fruit is often high up in the branches, making harvest challenging. The introduction of dwarfing rootstocks, that produce more manageable trees, makes the cultivation and harvest of cherry fruit easier due to the short stature of the plants (Table 2). However, cherry trees grown on dwarf rootstocks produce excessive numbers of small fruits with a low sugar content [176]. To overcome this, flower thinning (see Section 7.3) is undertaken to prevent over-yielding and to increase the quality of the fruit [176]. For a review, see Ruthowski and Lysiak [173] and Kurlus et al. [177]. Different rootstocks also provide advantages in different regions, (i.e., adapted for soil moisture content, soil types and soil temperatures), allowing the selection of rootstocks for the growing conditions.

In addition to these rootstocks (Table 2), new rootstocks are currently being commercialised, including Gisela 13 and 17, which have been described as vigorous, precocious, and productive; Weigel 3, described as semi-vigorous and adaptive to hot, dry conditions; and Krymsk 7, described as being the most vigorous of the Krymsk rootstocks to date. These new rootstocks currently require widespread evaluation. The requirement for newer rootstocks adapted to hotter, dryer conditions, such as Weigel 3, may be essential for protecting cherry cultivation from environmental change.

### 7.5. Protected Growth Environments

The introduction of dwarfing rootstocks and the use of biostimulants to initiate bud break, has also allowed for changes in methods of cultivation. In recent years, some cherry tree cultivators have started growing trees under cover in polytunnels, which provide protection from periodic weather extremes that can negatively impact yields, including late frosts [158]. Growth in polytunnels also protects the trees from excess rainfall (and rain-induced cracking), hail and from birds. Under extreme late season conditions, cultivators now use gas-powered heaters to prevent damaging frosts by propelling warm air 70 m along the polytunnel. Growth in a polytunnel also allows for unrestricted mechanical or hormonal “thinning” and fruit can be harvested irrespective of the weather. One drawback to this method of cultivation is the reduction or absence of wild pollinators, including solitary bees [25] inside the tunnel, thereby increasing reliance on domesticated honeybees (see Section 2.1.1).

## 8. Conclusions

Cherry is a nutritious and health-promoting fruit and worldwide production has increased by almost 40% in the last 20 years. These increases have been driven in part by a warming climate in parts of Europe, making the cultivation more advantageous, and by the introduction of dwarfing rootstocks and new methods of cultivation. However, cherry fruit contains a large stone and seed, the presence of which makes it a potential choking hazard for small children and inconvenient to eat ‘on the move’. Removal of the stone, through breeding or genetic manipulation, could be transformative for the cherry industry and facilitate rapid market expansion. The absence of the seeds/stone would also reduce processing costs, increase profits, and for the consumer, increases the potential of the fruit as a snacking product, therefore increasing consumer acceptance and consumption. An interesting model for stoneless fruit has been identified in *P. domestica* (Plum). This cultivar, known as ‘stoneless’, has been known in France for several hundred years and has been crossed with commercial varieties to transmit the stoneless trait. However, these results were not commercially viable [178], although segregation analysis suggested that the stoneless trait is linked to a single dominant gene [178]. Furthermore, RNA expression comparisons have shown that more than 2000 genes are differentially expressed when comparing ‘stoneless’ to normal stoned fruit, providing the first step to understanding the mechanism for the production of parthenocarpic fruit. Finally, parthenocarpic fruit has been identified in multiple species. Can this information provide a route to the development of a seedless cherry? Parthenocarpy has been reported in 96 Angiosperm taxa [179,180], and interestingly, almost half of all parthenocarpic species are trees. In particular, *Prunus persica* (L.) Batsch and *Prunus cerasifera* are the two woody plants with a drupe-type fruit in which parthenocarpy had been reported [179,180,181].

Over the last few decades, agricultural research has adopted technologies, such as genetic engineering and, more recently, ‘genome editing’ to improve traits in key crops [182,183,184,185,186]. There have been recent advances in the tools available to carry out this work, including vectors for multiple gene insertions [187,188,189,190,191] and tissue-specific promoters [192,193,194,195,196,197]. The generation of parthenocarpic fruit via genetic manipulation brings its own challenges. CRISPR-Cas9 genome editing has previously been used to generate parthenocarpic tomato plants, demonstrating the potential for this technology to manipulate seed development. However, fruit trees are the most reluctant species for in vitro tissue culture. In cherry, regeneration and transformation protocols have been created, using leaves, shoots and cotyledons as explants [198,199,200,201,202,203], suggesting that genome editing is a viable option for the generation of a stoneless, seedless fruit in cherry.

## Figures and Tables

**Table 1:** Some of the most commonly commercially cultivated varieties of sweet cherry. Early, mid and late ripening types based on the ripening of the reference variety, Burlat, a widespread cultivated cherry across Europe. Comparisons to the variety Bing are based on interviews with growers in Stanislaus County, California and on published data. NR—not reported.

Ripen	Days Post-Burlat *	Days Post Bing **	Variety	Reported Qualities and Region of Origin
Burlat	0	NR	Burlat	The most widespread and cultivated cherry among existing varieties. It has good flavor, high resistance to cracking compared to most varieties, and exceeds in its time.
Early	0–11	NR	Merchant	Self-sterile, mature 7 days post-Burlat. Producing good yields of large, dark red fruit in early summer. Self-sterile, mature 9 days after Burlat.
		NR	Carmen	Developed and cultivated in Hungary. Fruit have large size and good flavor. Spreading in Germany due to its high yields.
Mid	12–19	NR	Grace Star	Recent Italian variety. Self-fertile with maturation occurring 12 days post-Burlat. Large fruit, firm texture, excellent flavor qualities, and highly valued by consumers.
		−5	Cristalina^Tm^	Self-sterile cherry of Canadian origin. Mature 12 days post-Burlat. High productivity, under conditions of moderate load. Fruit have good firmness and very good flavor and is moderately sensitive to cracking.
		0	Stella	One of the first self-fertile varieties (Canadian origin). Ripens 19 days post-Burlat. Large fruit, blood-red hue, good resistance to cracking, sensitive to cold. Highly sought-after. Considered a cultivar of great importance.
		Bing	Bing	Most traditional and representative cherry of America. Ripening occurs 19 days post-Burlat. Fruit are dark red, large, firm, and highly valued for their excellent flavor and is the preeminent fresh-market cherry.
		0	Rainer	Bicolor cherry from the United States. The variety is self-sterile and ripens 19 days post-Burlat. Variety is appreciated by the industry owing to the large size and good flavor of the fruit.
Late	20–27	NR	Kordia	Originating in the Czech Republic with good resistance to cracking. Very popular in Germany. Ripens 24 days post-Burlat. Known for large, firm fruit and good flavor. Highly incompatible and setting virtually no fruit in the absence of cross-pollination.
		3	Sonata^Tm^	Self-fertile Canadian variety with good productivity. Ripens 22 days post-Burlat. Fruit have a good taste and are large and firm in size. Unfortunately, it is highly susceptible to cracking.
		5–7	Lapins	Highly productive self-fertile from the United States. Currently, most planted cherry in the world. Good flavor with cracking resistance. Variety is appreciated by farmers. Ripens 24 days post-Burlat (see Skeena^Tm^).
		7–10	Benton^Tm^	Another self-fertile cherry tree that ripens mid-season and has been reputed to surpass Bing cherries.
		10–14	Skeena^Tm^	Characterized as an improved Lapins. Similar characteristics, but lower productivity, which helps to produce cherries of greater caliber and quality. In the United States, replacing Lapins. Mature 25 days post-Burlat.
		11–13	Sweetheart^Tm^	Late maturation with large fruit. Prolific fruiters with a dark red, medium to large cherries. Pruning is required to keep trees productive.
Extra Late	More than 28	NR	Regina	Self-sterile variety of German origin. Low productivity with maturation occurring 31 days post-Burlat. Large sized fruit, good taste and cultivated due to its high resistance to cracking. Highly incompatible and setting virtually no fruit in the absence of cross-pollination.
		NR	Ambrunes	Spanish Cherry is traditionally grown in Cáceres. Firm fruit with very good flavor. High resistance to cracking. Ripens 31 days post-Burlat.

* https://en.excelentesprecios.com/cherry-tree-varieties, (3 June 2022); ** https://m.blog.naver.com/PostView.naver?isHttpsRedirect=true&blogId=2jw67&logNo=90164242596 1 June 2022. Tm = trademark.

**Table 2:** Some of the most commonly used Cherry rootstocks for commercially cultivated sweet cherry.

Rootstock	Reported Qualities of Dwafing Rootstocks
Mazzard F12.1	Original traditional *Prunus avium* cherry seedling rootstock used for centuries. Very compatible with most current cherry varieties. Produces a tree more than 20 ft in height. It is cold hardy, but not as hardy as Gisela rootstocks. Natural resistance to bacterial canker.
Mahaleb	A traditional seedling cherry rootstock. It produces a large slow-growing tree about 16 ft–20 ft tall. Suitable for a wide range of soils and suitable for drought conditions, but unsuitable for wet soils. Not compatible with all cherry varieties. Perfect for traditional orchards, although fruit may be difficult to harvest due to the height of the tree.
Gisela 5 (G5)	Originated from Justus Liebig University in Giessen, Germany in the 1960s. It produces a tree about 3 m/10 ft tall after 5 years. Has established itself as the rootstock of choice, producing a tree with manageable proportions. Trees cannot support themselves, requiring a permanent means of support (equivalent to the apple M26 rootstock). G6 can cause trees to flower early (by a few days), and have branches lower to the ground, increasing the likelihood of exposure to late frosts.
Gisela 6 (G6)	Produces a slightly larger tree than Gisela 5. An advantage over Gisela 5 is that it is much less fussy about soil conditions. Usually free-standing and does not need support (roughly equivalent to the apple MM106 rootstock). Suitable for a wide range of soil types, but good drainage is essential.
Gisela 12 (G12)	Produces a tree slightly more vigorous than Gisela 6. Gisela 12 is highly productive, self-supporting, and tolerant of most soils. Adapted to a range of soils.
Weigi 1	This sister rootstock is of Weigi 2. Semi-dwarf class, about 10% more vigorous than Gisela 5 (and Weigi 2). The rootstock of choice for poor soils and less vigorous varieties. Better productivity and larger fruit size than Gisela 5. Able to cope with high summer temperatures and a level of drought tolerance.
Weigi ^®^ 2	New semi-dwarf rootstock. This cherry tree is very similar in size to the Gisela 5. An advantage for UK growers is yield and fruit size are slightly improved compared to Gisela 5. More tolerant of drought and the higher summer temperatures found in southern Europe.
Krymsk 5	Widely used in commercial cherry orchards in the Pacific North West of the USA. Similar or slightly more vigorous than Gisela 6. Trees require no support. Not as productive as the Gisela, but fruit quality is good and trees are easier to manage. Developed specifically for cold-hardiness, but performs well in hot climates and heavy soils.
Krymsk 6	Developed from *Prunus cerasus* (the sour cherry). It is a semi-dwarf rootstock, suitable for hot and cold climates.
Colt	Developed at East Malling Research Station in Kent in 1977. Cross between the sweet cherry *Prunus avium* and the less vigorous *Prunus pseudocerasus*. First dwarfing rootstock for sweet cherries. Considered the best rootstock for growing trees in large gardens and community orchards. The tree has a height of 12–15 ft and tolerates poorer soils better than Gisela 5 and needs less looking after (comparable to the apple MM111 rootstock and the PyroDwarf pear rootstock). Colt is resistant to phytophthora root rot, bacterial canker and stem-pitting, but is more susceptive to crown gall.

https://www.orangepippintrees.co.uk/articles/fruit-tree-gardening/rootstocks-for-cherry-trees 1 June 2022. https://www.goodfruit.com/a-lot-of-choices-for-cherry-rootstocks/, 1 June 2022.

## Data Availability

Not applicable.
